# What’s new in Emergencies, Trauma, and Shock? Developing predictor models for infectious diseases

**DOI:** 10.4103/0974-2700.66518

**Published:** 2010

**Authors:** Takashi Kokudo, Fukumi Nakamura-Uchiyama, Kenji Ohnishi

**Affiliations:** Department of Infectious Diseases, Tokyo Metropolitan Bokutoh General Hospital, 4-23-15 Koutobashi, Sumida-ku, Tokyo 130-8575, Japan

Leptospirosis is a zoonotic disease caused by infection with pathogenic spirochetes of the genus *Leptospira*. The disease is maintained in nature by the chronic renal infection of animals, mainly rodents. Since transmission occurs via contact with urine from infected animals, veterinarians, sewer workers, farmers, ranchers, abattoir workers, trappers, loggers, and rice field workers are at a high risk of exposure to *Leptospira*. The disease is endemic in tropical regions and extremely rare in developed countries. However, there are still several reports showing that leptospiral infection can occur in urban areas.[1]

Leptospiral infection is associated with a broad spectrum of severity. Signs and symptoms of leptospirosis are generally mild; however, around 5% of cases develop a severe form of leptospirosis (i.e. Weil’s disease).[2] So, many clinicians, those working not only in developing countries but also in developed countries, are eager to have a scoring system to predict the severity and mortality of leptospirosis. The clinical course of Weil’s disease can be divided into three stages: febrile stage, icteric stage, and convalescent stage. Since high fever and headaches are the most frequent symptoms in the febrile stage, making it difficult to distinguish from other infectious diseases, most cases of Weil’s disease are diagnosed in the following icteric stage. This fact makes accurate assessment of the disease burden and risk for poor prognosis difficult.

Prediction models for severity and mortality in infectious diseases have been previously discussed, especially in sepsis. The first concept used for sepsis was systemic inflammatory response syndrome (SIRS).[3] However, a stricter model was necessary, and the predisposition, infection, response, organ dysfunction (PIRO) model was suggested.[4]

In the *Journal of Emergencies, Trauma, and Shock*, the authors reviewed the literature and identified several prognostic indicators of leptospirosis according to the PIRO model, for the first time.

Increasing age and chronic alcoholism were predisposing risk factors. Although the exact mechanism is unclear, in severe leptospirosis, jaundice is a frequent symptom and this may be related to chronic alcoholism. Severe jaundice with little or no elevation of hepatobiliary enzymes is characteristic in leptospirosis, and in the histopathology, the overall structure of the liver is not significantly disrupted. However, intrahepatic cholestasis, hypertrophy and hyperplasia of Kupffer cells, as well as erythrophagocytosis have been reported.[2] The main etiology of jaundice is not considered to be related to liver necrosis and dysfunction; however, chronic alcoholism, being a risk factor for morbidity, may suggest that background liver function may be important for the severity of leptospirosis.

Leptospiremic burden rather than the infected serovars is a risk factor. In Weil’s disease, leptospires are present in blood only in the febrile stage. This means that leptospires are rarely present in the icteric stage when the symptoms are most prominent. It is also reported that serovars which develop Weil’s disease are *L. copenhageni* and *L. icterohaemorrhagiae*.[1] However, there was no relationship observed between morbidity and infecting serovars. This may help us to understand the etiology of severe leptospirosis and needs further investigation.

Hemodynamic disturbances, leukocytosis, multiple organ failure, renal involvement, and pulmonary involvement were also identified as risk factors. Renal involvement is one of the triads of Weil’s disease and sometimes needs transient hemodialysis. Regarding pulmonary involvement, there are several reports that pulmonary hemorrhage was critical in severe leptospirosis.[5] Since the clinical course of leptospirosis is sometimes biphasic, the stage in which we evaluate the response and organ failure factors for prognosis is important. If there are applicable prognostic factors in the early stage, it would be very useful in clinical practice.

Although evaluating risk factors is very clear according to the PIRO model, there are several factors that should be considered in leptospirosis, which cannot be classified in this model. They are mainly treatment related. Daily dialysis had a survival advantage over alternate day dialysis and peritoneal dialysis for patients with acute renal failure. Antibiotic treatment has conflicting results in leptospirosis. Although patients with severe disease were excluded from the study, doxycycline (100 mg twice a day for 7 days) was shown to reduce the duration and severity of illness in anicteric leptospirosis.[6] Intravenous penicillin (8 MU/day for 5 days) showed no difference in outcome or duration of the illness between treatment and control groups.[7] However, further investigation is necessary for antibiotic treatment.

Because of the differences in method and non comparability of data in the literature which the authors reviewed, they could not make a clear scoring system like that of the PIRO model for sepsis. The fact that the clinical course of leptospirosis is sometimes biphasic and has regional characteristics makes a prospective study with a large sample size difficult to perform. Since the febrile stage is difficult to distinguish from other infectious diseases, and physicians, especially those working in urban areas, are unfamiliar with this disease, many cases are found in the icteric stage. However, early stage factors to predict the severity of leptospirosis are hoped for and a study designed for this purpose is necessary.

Although the authors could not make a clear staging system, this report could be a milestone for predicting the mortality and severity of leptospirosis according to the PIRO model.
